# Brachydactyly Type A1 Caused by an *IHH* Variant in a Patient with Disproportionate Short Stature: A Case Report

**DOI:** 10.3390/reports9030294

**Published:** 2026-09-01

**Authors:** Inés García de Pablo, María Cristina Ontoria Betancort, Francisco Martínez Bugallo, Sebastián Eustaquio Martín Pérez, Isidro Miguel Martín Pérez

**Affiliations:** 1 Servicio de Pediatría, Hospital Universitario Nuestra Señora de Candelaria, 38010 Santa Cruz de Tenerife, Santa Cruz de Tenerife, Spain; 2 Unidad de Genética, Servicio de Análisis Clínicos, Hospital Universitario Nuestra Señora de Candelaria, 38010 Santa Cruz de Tenerife, Santa Cruz de Tenerife, Spain; 3 Faculty of Health Sciences, Universidad Europea de Canarias, 38300 La Orotava, Santa Cruz de Tenerife, Spain; 4 Faculty of Medicine, Health and Sports, Universidad Europea de Madrid, 28670 Villaviciosa de Odón, Madrid, Spain; 5 Faculty of Health Sciences, Universidad del Atlántico Medio, 35017 Tafira Baja, Las Palmas, Spain; 6 Hospital Universitario Vithas Las Palmas, 35005 Las Palmas de Gran Canaria, Las Palmas, Spain

**Keywords:** short stature, brachydactyly type A1, *IHH* gene, skeletal dysplasia, clinical exome sequencing, growth hormone deficiency, growth hormone therapy

## Abstract

**Introduction and Clinical Significance:** Skeletal dysplasias comprise a genetically heterogeneous group of disorders with substantial phenotypic overlap, often complicating diagnosis. Clinical exome sequencing (CES) can facilitate molecular diagnosis in children with unexplained disproportionate short stature. **Case Presentation:** An 8-year-old boy presented with severe short stature (−3.24 SDS), brachydactyly, relative macrocephaly, broad nasal bridge, and mild calf hypertrophy. Endocrine evaluation confirmed growth hormone deficiency (GHD). Following negative *SHOX* testing, CES identified a heterozygous likely pathogenic *IHH* variant (c.446G>A; p.Arg149His), establishing the diagnosis of brachydactyly type A1 (BDA1). Recombinant human growth hormone (rhGH), initiated for GHD, resulted in improved growth velocity and height SDS. Transient unilateral prepubertal gynecomastia developed during treatment and resolved after temporary rhGH withdrawal, with no recurrence following reinitiation. **Conclusions:** This case highlights the diagnostic value of CES in children with disproportionate short stature after unrevealing targeted testing and illustrates that GHD may coexist with *IHH*-related skeletal dysplasia. An integrated genetic and endocrine evaluation can refine diagnosis, identify coexisting treatable endocrine disorders, and guide individualized management.

## 1. Introduction and Clinical Significance

During childhood, linear growth is a regulated process involving the coordinated interplay of endocrine, genetic, nutritional, and environmental factors. Longitudinal bone growth takes place at the growth plate (physis), where chondrocyte proliferation, differentiation, and hypertrophy are precisely regulated by complex molecular signaling pathways [1]. Disruption of these mechanisms can impair endochondral ossification and result in a broad spectrum of disorders, ranging from isolated short stature to severe skeletal dysplasias. Their marked genetic heterogeneity and variable clinical expression often make these conditions challenging to recognize in pediatric practice [2].

Skeletal dysplasias comprise a heterogeneous group of more than 460 inherited disorders affecting bone and cartilage [3]. Although individually rare, they collectively represent a relevant cause of disproportionate short stature and skeletal abnormalities. Early diagnosis can be difficult because children may initially present with nonspecific features, such as impaired growth, brachydactyly, limb shortening, or delayed skeletal maturation, whereas characteristic radiographic findings may become evident only as development progresses [4,5,6]. This age-dependent phenotypic evolution can frequently delay the appropriate diagnosis and management, genetic counselling, and therapeutic decision-making.

Additionally, accurate diagnosis requires the integration of clinical, radiographic, and molecular genetic findings. Increasing evidence has highlighted the central role of genes involved in growth plate homeostasis and chondrocyte differentiation in syndromic and non-syndromic growth disorders [7,8]. Therefore, the early identification of a pathogenic variant not only establishes the molecular diagnosis but may also accelerate diagnosis, improve prognostic assessment, facilitate genetic counselling, and guide individualized management. Among recent genetic studies, *SHOX* analysis is recommended as a first-line test in children presenting disproportionate short stature, as *SHOX* deficiency represents one of the most common monogenic causes of impaired linear growth [9].

However, when *SHOX* testing is negative, the subsequent diagnostic approach is less straightforward [10]. Traditionally, this has involved sequential testing of candidate genes selected according to the clinical phenotype [11]. Although this strategy may be effective in patients with highly characteristic features, its diagnostic yield is still limited by the coexistence of phenotypic overlap and variable expressivity of childhood skeletal dysplasias [12,13]. Next-generation sequencing (NGS) approaches, particularly clinical exome sequencing, have become increasingly useful by enabling the simultaneous analysis of multiple genes involved in skeletal development and growth plate function, thereby improving diagnostic efficiency.

These challenges are relevant in disorders affecting endochondral ossification, such as hypochondroplasia and brachydactyly type A1 (BDA1). Hypochondroplasia is caused by pathogenic variants in Fibroblast growth factor receptor 3 (*FGFR3*) and is typically characterized by short stature, mesomelic limb shortening, brachydactyly, broad hands, and increased muscular bulk [14,15,16]. Conversely, BDA1 is caused by heterozygous pathogenic variants in Indian Hedgehog (*IHH*), a key regulator of growth plate organization and chondrocyte differentiation, and is characterized by shortening or aplasia of the middle phalanges [17,18,19,20].

Furthermore, biallelic *IHH* variants are associated with acrocapitofemoral dysplasia (OMIM #607778), a rare skeletal dysplasia characterized by a disproportionate short stature, brachydactyly, and abnormal epiphyseal development [21]. Despite their distinct molecular etiologies, *IHH*-related disorders may exhibit overlapping clinical and radiographic features, particularly in early childhood, which may complicate their recognition and differential diagnosis. Importantly, little is known about the coexistence of *IHH*-related skeletal disorders with endocrine causes of growth impairment, and their combined impact on growth remains insufficiently characterized [22].

Growth hormone deficiency (GHD), when present, may further complicate the clinical assessment, as both skeletal dysplasia and GHD can contribute to impaired growth velocity and reduced adult height [23]. In addition, evidence regarding the efficacy and longitudinal response to recombinant human growth hormone (rhGH) in patients harboring pathogenic *IHH* variants remains scarce, leaving an important gap in their clinical management [24,25].

Clinical exome sequencing has emerged as a valuable diagnostic approach when conventional clinical, endocrine, radiographic, and targeted genetic investigations fail to establish a definitive diagnosis. In this context, and given the limited evidence regarding the coexistence of *IHH*-related skeletal disorders and endocrine disorders of growth impairment, we report a boy with disproportionate short stature and negative *SHOX* testing, initially suspected of having another skeletal dysplasia, in whom exome sequencing identified a pathogenic *IHH* variant consistent with BDA1, together with concomitant GHD. This rare association highlights the clinical value of genomic testing in children with unexplained disproportionate short stature and expands the limited evidence on growth response to rhGH in patients with coexisting IHH-related skeletal dysplasia and GHD.

## 2. Case Presentation

### 2.1. Patient Information

An 8-year-old boy was referred to the Pediatric Endocrinology Unit at Hospital Universitario Nuestra Señora de Candelaria (HUNSC), Tenerife, Spain, for evaluation of severe disproportionate short stature and impaired linear growth. Longitudinal growth records showed a decline in height percentile from approximately 3 months of age, followed by relative stabilization between 6 and 8 months of age. Thereafter, his height remained consistently below the expected range for age and sex and substantially below his target height throughout childhood.

Additionally, he was born at term following an uncomplicated pregnancy and spontaneous vaginal delivery. The neonatal period was challenged by the existence of a meconium aspiration syndrome (MAS), requiring a brief hospital admission, with a complete recovery and no subsequent clinical sequelae. Developmental milestones were achieved at the expected ages, and his subsequent medical history was unremarkable, with no previous surgery or known allergies. Routine neonatal screening and childhood immunizations were fully completed according to national recommendations. Serial growth monitoring revealed persistent impairment of linear growth, which ultimately prompted referral for specialist evaluation. No specific treatment for short stature had been initiated before referral.

Family history revealed clustering of short stature on the maternal side. The mother measured 147.2 cm (<P1; −2.83 SDS), whereas the father measured 185 cm (P88; +1.18 SDS), resulting in a target height of 172.6 ± 5 cm (P23; −0.76 SDS). Several maternal relatives, including the mother’s siblings, also showed short stature. Clinical assessment of the mother showed shortening of the fourth metacarpals and metatarsals, particularly in the feet, suggesting an autosomal dominant pattern of inheritance. No parental consanguinity was reported.

Furthermore, molecular analysis, performed at the Genetics Unit, Department of Clinical Analysis, Hospital Universitario Nuestra Señora de Candelaria, identified a heterozygous *IHH* variant (c.446G>A; p.Arg149His), establishing the diagnosis of BDA1. In addition, the patient fulfilled the diagnostic criteria for GHD, supporting the coexistence of two distinct conditions contributing to impaired linear growth. This report was approved by the Ethics Committee of Hospital Universitario Nuestra Señora de Candelaria (CHUNSC_2025_19; 6 March 2025), and written informed consent was obtained from the patient’s father.

### 2.2. Clinical Findings

At presentation, the patient exhibited marked disproportionate short stature, with height persistently below −3.23 SDS (<P1) for age and sex and substantially below his target height (P23; −0.76 SDS). Growth velocity was reduced (4.1 cm/year; P6; −1.62 SDS), while his body mass index (BMI) remained within the normal range. Anthropometric assessment confirmed significant body disproportion, with a reduced arm span relative to height and an increased sitting height-to-height ratio of 0.569 (P83), indicating relative shortening of the lower limbs. These findings raised strong suspicion of an underlying skeletal dysplasia.

Subsequently, physical examination was notable for a relatively large head in proportion to body size, a broad and flattened nasal bridge, high-arched palate, dental malocclusion, muscular habitus with mild bilateral calf hypertrophy, and a solitary café-au-lait macule on the left cheek. No cubital bowing, Madelung deformity, or other evident skeletal abnormalities were identified. Inspection of the hands showed bilateral brachydactyly, predominantly affecting the IV and V rays. Cardiovascular, abdominal, neurological, and systemic examinations were unremarkable; however, he reported mild, self-limited headaches without associated neurological manifestations.

At the most recent follow-up, at 11 years and 6 months of age, height had increased from 113.9 to 129.8 cm, corresponding to an improvement in height SDS from −3.23 to −2.65, although height remained below the 1st percentile. Growth velocity improved from 4.1 cm/year (P6; −1.62 SDS) to 4.9 cm/year (P55; +0.15 SDS), supporting a favorable response to rhGH therapy. Over the same period, weight increased from 23.7 to 36.0 kg, while BMI remained within the normal range (21.37 kg/m^2^; P70; +0.55 SDS). The patient remained prepubertal (Tanner stage I), with bilateral testicular volumes of 3 mL.

### 2.3. Timeline

Growth impairment became evident during childhood, with a progressive decline in height percentile from approximately 3 months of age. At the age of 8, the patient presented with severe short stature (height, 113.9 cm; −3.23 SDS), reduced growth velocity (4.1 cm/year; −1.62 SDS), and persistent body disproportion, reflected by a reduced arm span relative to height and an increased sitting height-to-height ratio (0.569; P83). Given these findings, SHOX analysis was carried out as the first-line genetic investigation but was negative. Further endocrine evaluation, including two additional growth hormone (GH) stimulation tests with clonidine and glucagon, showed subnormal peak GH responses, confirming the diagnosis of GHD.

**Figure 1:**
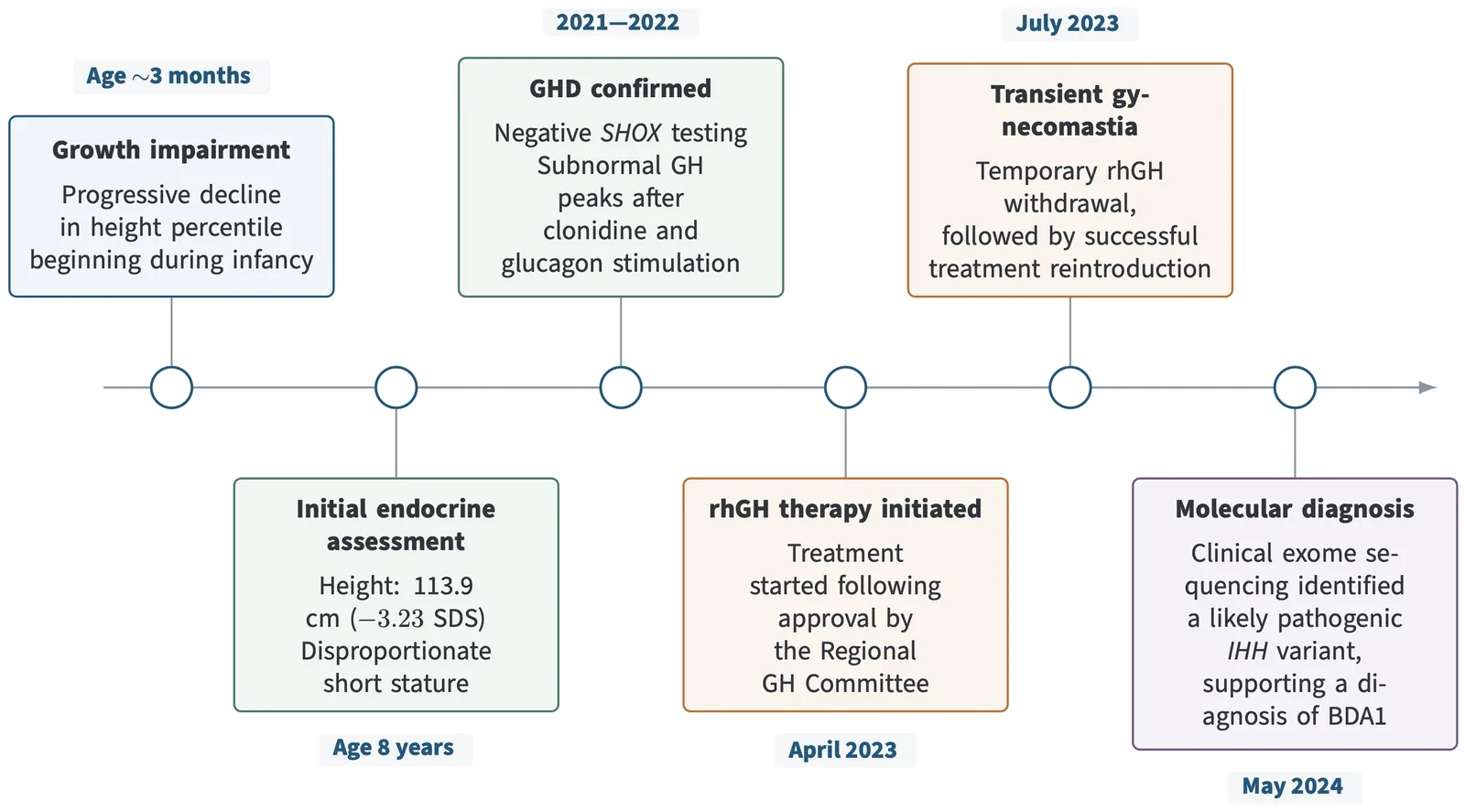
Clinical timeline showing growth impairment, GHD diagnosis, rhGH therapy, transient gynecomastia, and molecular diagnosis by clinical exome sequencing. Abbreviations: BDA1, Brachydactyly Type A1; GHD, growth hormone deficiency; GH, growth hormone; *IHH*, Indian hedgehog; rhGH, recombinant human growth hormone; SDS, standard deviation score. Source: own work.

Subsequently, rhGH therapy was initiated in April 2023 following approval by the Regional Growth Hormone Committee. Three months later, transient unilateral right-sided prepubertal gynecomastia developed, resolving completely after temporary treatment withdrawal; rhGH was subsequently resumed without recurrence. Persistent body disproportion and brachydactyly despite GHD prompted genetic evaluation. Clinical exome sequencing in May 2024 identified a heterozygous likely pathogenic *IHH* variant (NM_002181.4:c.446G>A; p.Arg149His), confirmed by Sanger sequencing, establishing the diagnosis of BDA1 and explaining the skeletal phenotype (Figure 1).

### 2.4. Diagnostic Assessment

Comprehensive auxological assessment included measurements of height, weight, BMI, arm span, sitting height, and sitting height-to-height ratio. Baseline laboratory evaluation included a complete blood count, serum biochemistry, thyroid function tests, celiac disease screening, cortisol, sex steroids, IGF-1, and IGFBP-3, all of which were within age-adjusted reference ranges. Furthermore, endocrine evaluation confirmed GHD, with peak GH concentrations of 5.1 ng/mL after clonidine and 5.19 ng/mL after glucagon stimulation, both below the laboratory-specific diagnostic cut-off of 7 ng/mL. Baseline IGF-1 was 121 ng/mL (−0.8 SDS) (Table 1).

**Figure 2:**
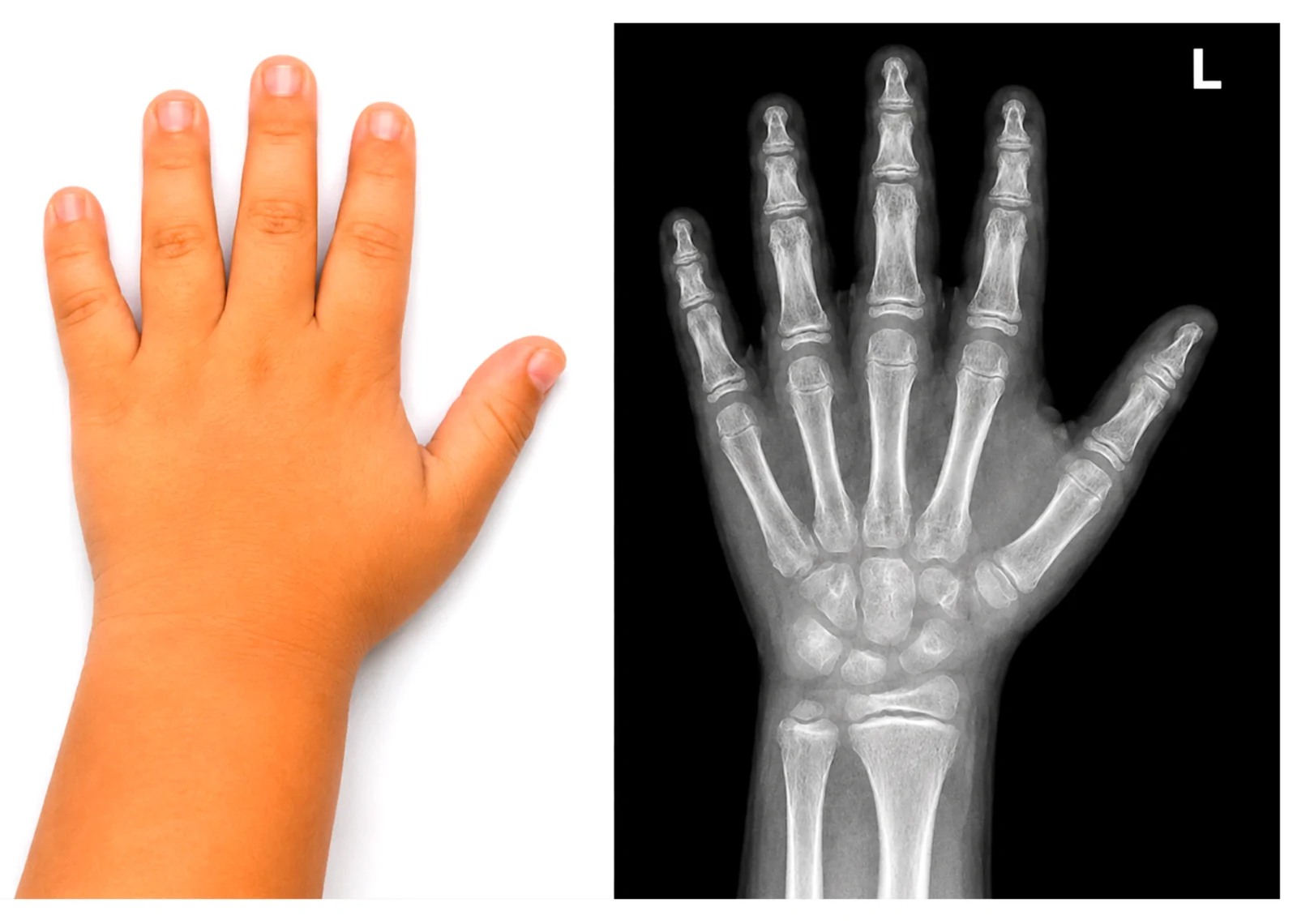
Clinical and radiographic appearance of the left hand. Dorsal image of the left hand (**left**) and its corresponding PA-HW X-ray (**right**). The images show shortening of the middle phalanges and brachydactyly, findings consistent with BDA1 linked with a pathogenic *IHH* variant. Radiographic evaluation using GPA showed delayed skeletal maturation relative to CA. Source: own work.

Additionally, bone maturation was assessed using a left posteroanterior hand and wrist (PA-HW) X-ray according to the Greulich and Pyle Atlas (GPA), developed and validated for the pediatric population of the Canary Islands [26,27,28,29]. Radiographic assessment revealed delayed skeletal maturation, with a bone age (BA) of 5–5.5 years at a chronological age (CA) of 7 years and 5 months (Figure 2).

Furthermore, karyotyping showed a normal 46,XY karyotype, while *SHOX* analysis yielded negative results. Given the persistent clinical suspicion of an underlying skeletal dysplasia, clinical exome sequencing was performed using the Agilent Constitutional Panel (17 Mb), targeting more than 5600 genes associated with inherited disorders. Genomic DNA was extracted from peripheral blood using the MagCore^©^ Genomic DNA Whole Blood Kit. Sequencing libraries were prepared using Agilent SureSelect^™^ capture technology and sequenced on a NextSeq 550 System. Sequence reads were aligned to the GRCh37/hg19 human reference genome and subsequently subjected to variant calling, annotation, and filtering based on different criteria such as allele frequency, predicted functional impact, inheritance pattern, and phenotype–genotype correlation.

For variant assessment, population and clinical databases, including the Genome Aggregation Database (gnomAD), ClinVar, Human Gene Mutation Database (HGMD), and Leiden Open Variation Database (LOVD), were queried alongside in silico prediction tools, including Combined Annotation Dependent Depletion (CADD) and Rare Exome Variant Ensemble Learner (REVEL). Thereafter, variants were classified according to the ACMG/AMP criteria. Molecular analysis identified the heterozygous *IHH* variant NM_002181.4:c.446G>A [p.(Arg149His)], previously associated with BDA1. The variant was absent from gnomAD and had not been reported in ClinVar or LOVD, while in silico prediction tools, including CADD and REVEL, supported the existence of a deleterious effect. Based on the collected evidence, the detected variant was classified as likely pathogenic and subsequently confirmed by Sanger sequencing.

The differential diagnosis included *SHOX* haploinsufficiency, hypochondroplasia, pseudohypoparathyroidism (PHP), acrodysostosis (ACRD), ACAN- and NPR2-related short stature, GDF5-related brachydactyly, and trichorhinophalangeal syndrome (TRPS). These disorders were considered less consistent with the patient’s overall clinical and radiographic phenotype and the observed familial pattern. In particular, PHP was deemed unlikely given the normal calcium and phosphate levels and the absence of biochemical evidence of mineral metabolism abnormalities or hormone resistance. In the case of ACRD, and the remaining differential diagnoses were likewise considered less compatible with the overall clinical, biochemical, and radiographic findings and the familial pattern. In contrast, the characteristic pattern of brachydactyly, combined with the identification of a likely pathogenic *IHH* variant provided compelling phenotypic and molecular evidence for a diagnosis of BDA1 (OMIM #112500), coexisting with GHD and familial short stature.

### 2.5. Therapeutic Intervention

Following assessment and approval by the Regional Growth Hormone Committee, rhGH therapy was initiated in April 2023 at a subcutaneous dose of 0.7 mg/day (approx. 30 μg/kg/day). Approximately three months later, the patient developed transient unilateral right-sided prepubertal gynecomastia. A comprehensive evaluation, including hormonal profiling, tumor markers, and breast ultrasonography, excluded endocrine, structural, and neoplastic causes. Given the temporal association with rhGH therapy, treatment was temporarily discontinued as a precaution.

Breast enlargement resolved almost completely within two months of rhGH withdrawal. After repeat clinical and endocrine evaluation, treatment was resumed at the original dose, with no recurrence of gynecomastia or other treatment-related adverse events. Thereafter, the dose was adjusted according to weight, growth response, and biochemical parameters, maintaining an average of 27–30 μg/kg/day.

### 2.6. Follow-Up and Outcomes

**Figure 3:**
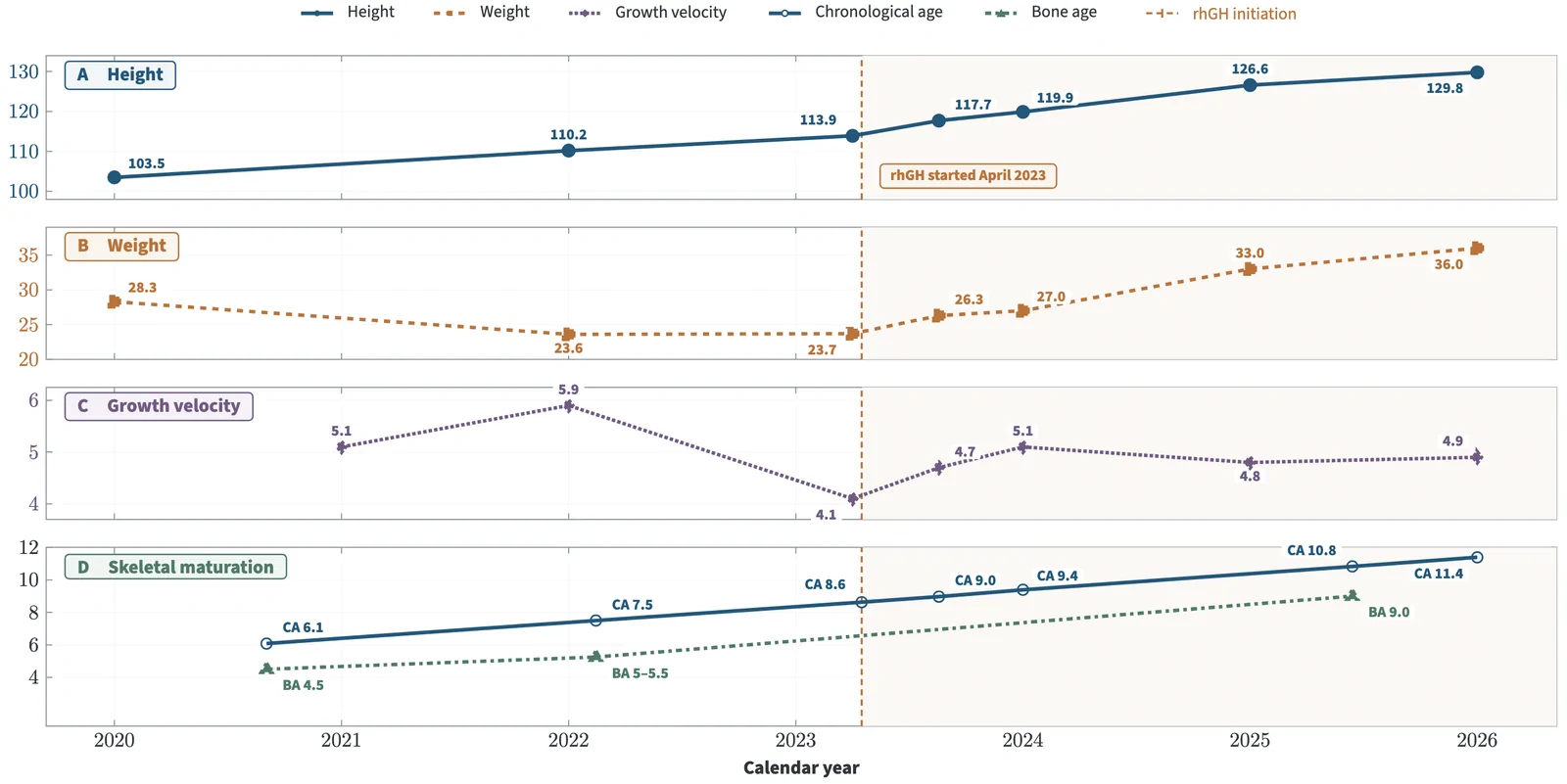
Height (blue solid line), weight (orange dashed line), growth velocity (violet dotted line), chronological age (CA; blue solid line with open circles), and bone age (BA; green dash-dotted line with triangles) are shown throughout follow-up. The orange dashed vertical line indicates the initiation of rhGH therapy in April 2023, and the shaded area represents the treatment period. Serial BA assessments show persistent delayed skeletal maturation relative to CA.

During rhGH therapy, follow-up was performed every six months and included standardized auxological assessment (height, weight, BMI, growth velocity, and height SDS), serum IGF-1 and biochemical monitoring, and serial BA assessment. Growth velocity increased from 3.9 cm/year (P3; −2.05 SDS) before treatment to 5.1 cm/year in 2024, 4.8 cm/year in 2025, and 4.9 cm/year (P55; +0.15 SDS) in 2026. Over the same period, height SDS improved from −3.23 at treatment initiation to −3.06, −2.89, and −2.65, corresponding to annual gains of +0.17, +0.17, and +0.24 SDS, respectively. Despite persistent short stature, the patient remained prepubertal at 11 years and 6 months (testicular volume, 3 mL), indicating substantial residual growth potential (Figure 3).

Biochemical monitoring showed proper IGF-1 response within the upper-normal range, with persistent mild BA delay relative to CA. Treatment was well tolerated, with no evidence of intracranial hypertension, impaired glucose metabolism, thyroid dysfunction, or other clinically relevant adverse events. The only notable adverse event during rhGH treatment was transient unilateral prepubertal gynecomastia, confirmed by ultrasonography (right breast, 4.6 × 18 mm; left breast, 2.5 × 5.4 mm), which resolved after temporary rhGH withdrawal and did not recur following treatment reintroduction.

At the latest follow-up, growth velocity and biochemical parameters remained stable, with no additional adverse events or clinical signs of osteoarticular complications, including slipped capital femoral epiphysis. Importantly, in this patient with BDA1 and concomitant GHD, rhGH therapy was associated with sustained improvement in linear growth and a favorable safety profile.

## 3. Discussion

Pathogenic variants in the *IHH* gene disrupt endochondral ossification and give rise to a phenotypic spectrum ranging from autosomal dominant BDA1 (OMIM #112500) to autosomal recessive acrocapitofemoral dysplasia (OMIM #607778). Most reported pathogenic variants cluster within the biologically active amino-terminal region of the *IHH* protein [30]. In our patient, the combination of short broad phalanges, short stature, an increased sitting height-to-height ratio, reduced arm span, and familial short stature was consistent with an *IHH*-related skeletal dysplasia [31]. Beyond establishing the molecular diagnosis of BDA1, this case is clinically noteworthy because of the coexistence of GHD, for which rhGH therapy was indicated, a favorable response to treatment, and transient gynecomastia during follow-up.

This case also illustrates the diagnostic challenges associated with disproportionate short stature. Phenotypic overlap among skeletal dysplasias may delay the molecular diagnosis, particularly when a sequential gene-by-gene approach is used [32]. The differential diagnosis included PHP and ACRD, both of which may present with skeletal abnormalities and endocrine manifestations. PHP was considered unlikely because calcium and phosphate concentrations were normal and no evidence of altered mineral metabolism or hormone resistance was observed [33,34]. ACRD was also considered, however, the clinical and radiographic phenotype and familial pattern were more consistent with BDA1 [35]. Other genetic causes of disproportionate short stature and brachydactyly, including ACAN-, NPR2-, and GDF5-related disorders and TRPS, were also considered. Nevertheless, the phenotypic and radiographic pattern, together with the apparent autosomal dominant familial presentation, favored BDA1 [36,37].

Increasing evidence supports the early use of NGS in children with short stature, brachydactyly, or a suggestive family history. In our patient, negative *SHOX* testing prompted broader molecular analysis, which identified the heterozygous *IHH* variant NM_002181.4:c.446G>A [p.(Arg149His)], subsequently confirmed by Sanger sequencing. The molecular finding and the clinical and radiographic features supported the final diagnosis of BDA1 and resolved the previous diagnostic uncertainty [38]. Although segregation analysis was not performed, the family history was notable for short stature in the mother and other maternal relatives.

Regarding variant interpretation, the *IHH* c.446G>A [p.(Arg149His)] variant was classified as likely pathogenic according to the ACMG/AMP framework. It was absent from the gnomAD population database and was not recorded in ClinVar or *LOVD*. Evidence supporting its classification included PM2_Strong (+4), PM1_Supporting (+1), PP2 (+1), and PP3 (+1), with the latter supported by concordant in silico predictions from CADD and REVEL suggesting the existence of a deleterious effect [39,40,41]. Consistent with our patient’s phenotype, the *IHH* c.446G>A [p.(Arg149His)] variant has previously been identified in individuals with short stature and brachydactyly [42,43].

Additionally, the same variant was identified in two unrelated patients assessed at our Unit who exhibited a highly concordant phenotype characterized by short stature, an increased sitting height-to-height ratio, and brachydactyly. Despite the lack of functional studies of p.(Arg149His), its rarity in the general population, computational predictions, and the phenotypic concordance observed across those affected support its classification as likely pathogenic. Familial segregation analysis was not performed in the present patient, therefore, the parental origin of the variant remains unknown, representing a limitation of this report. Targeted testing of first-degree relatives could provide additional segregation evidence and further inform genetic counseling.

The coexistence of GHD and an *IHH*-related disorder is particularly noteworthy because endocrine abnormalities are not currently recognized as a typical component of the *IHH*-related phenotype [42,44]. In our patient, GHD was supported by subnormal peak GH responses on two independent stimulation tests, whereas thyroid and adrenal function were preserved and gonadal hormone concentrations were consistent with prepubertal status (TSH, 2.14 mIU/L; free T4, 1.21 ng/dL; morning cortisol, 14.8 µg/dL; LH, 0.12 IU/L; FSH, 0.84 IU/L; testosterone, <0.05 ng/mL).

Chen et al. [45] similarly described two siblings harboring an *IHH* variant who exhibited subnormal GH responses and substantial improvement in linear growth during four years of rhGH therapy. However, GH stimulation tests have recognized limitations in specificity and reproducibility, and the evidence remains insufficient to establish a causal relationship between *IHH* dysfunction and impaired GH secretion [46,47,48]. GHD and *IHH*-related skeletal dysplasia should therefore currently be regarded as potentially coexisting conditions and evaluated independently.

In this patient, rhGH therapy was initiated exclusively for confirmed GHD and not for BDA1 or the *IHH* variant itself, as rhGH is not an approved treatment for BDA1 or dysplasia per se. The favorable auxological response indicates preserved responsiveness to the GH/IGF-1 axis despite the underlying growth-plate disorder. Together with the observations reported by Chen et al. [45], our findings suggest that the presence of an *IHH*-related skeletal dysplasia does not necessarily preclude a clinically meaningful response to rhGH when an established therapeutic indication, such as GHD, is present. Importantly, these findings should not be interpreted as supporting rhGH therapy for BDA1 in the absence of GHD or another approved indication. Further studies are needed to clarify whether *IHH* variants may be associated with altered GH secretion in a subset of patients and to characterize the long-term response to rhGH in patients with concomitant GHD and *IHH*-related skeletal dysplasia.

A further notable finding was transient unilateral prepubertal gynecomastia during rhGH therapy. Although uncommon, gynecomastia has previously been reported in association with GH treatment [49,50,51,52]. In our patient, the temporal relationship with rhGH exposure and resolution following temporary treatment withdrawal raised the possibility of a treatment-related association. However, the absence of recurrence after successful rhGH reintroduction precludes a firm causal inference. This clinical course supports an individualized approach in which potentially treatment-related adverse events are carefully investigated while avoiding unnecessary permanent discontinuation when therapy remains clinically indicated. Given the underlying skeletal dysplasia, continued surveillance for musculoskeletal complications, particularly slipped capital femoral epiphysis, is warranted during the application of rhGH therapy. Bone mineral density was not systematically assessed, therefore, osteopenia cannot be formally excluded and represents a limitation of this report [53,54].

**Figure 4:**
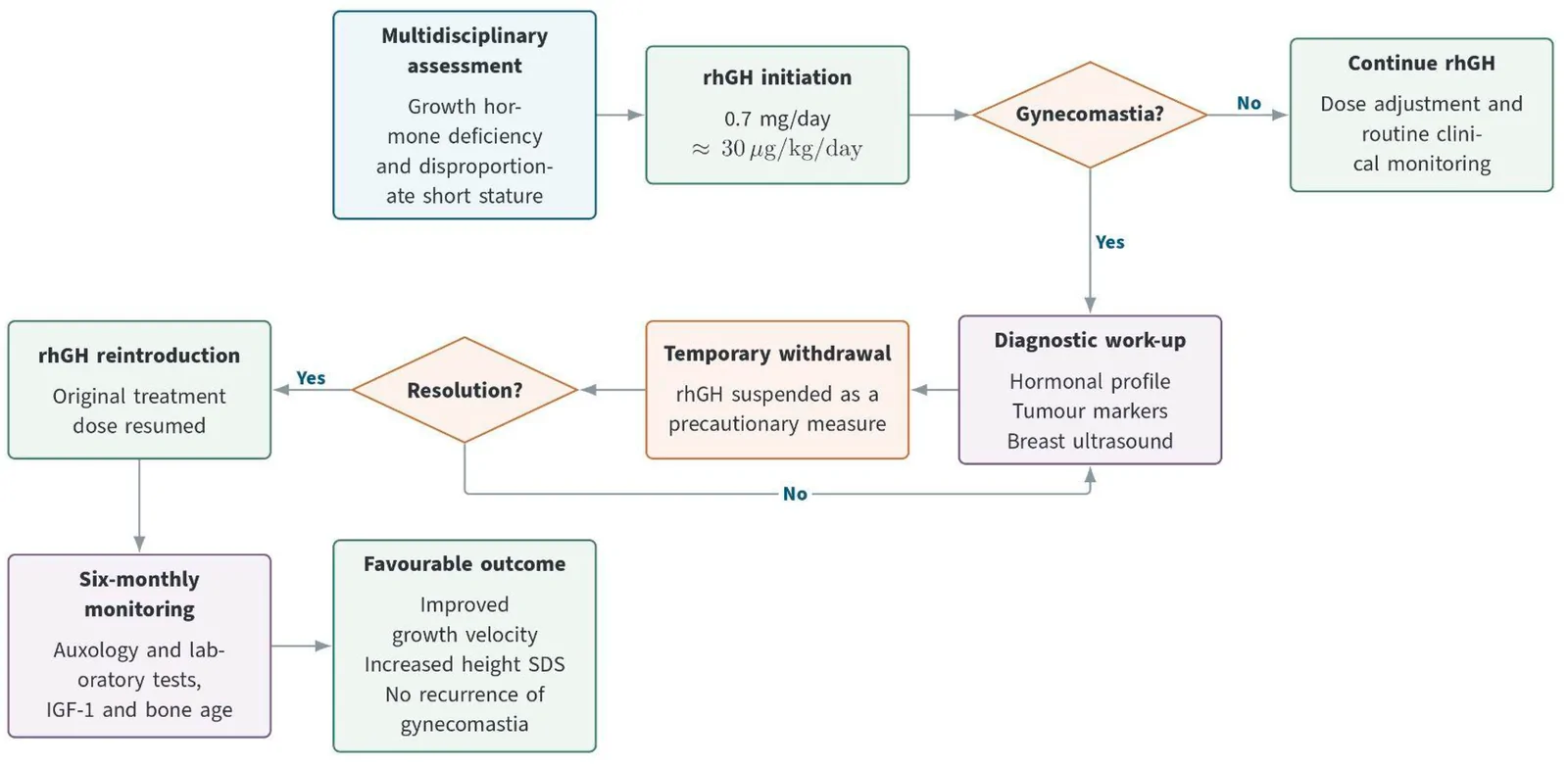
Clinical management algorithm illustrating the therapeutic pathway for rhGH treatment, including assessment, treatment initiation, diagnosis of gynecomastia, temporary treatment withdrawal, rhGH reintroduction after symptom resolution, and long-term clinical follow-up. Abbreviations: GH, growth hormone; IGF-1, insulin-like growth factor 1; rhGH, recombinant human growth hormone; SDS, standard deviation score. Source: own work.

Overall, this case highlights several clinically relevant considerations. First, early molecular testing should be considered in children with disproportionate short stature, brachydactyly, characteristic skeletal findings, or a positive family history, particularly when targeted genetic testing is unrevealing. Second, the diagnosis of skeletal dysplasia should not preclude evaluation for a coexisting and potentially treatable endocrine disorder. Third, when rhGH therapy is indicated for confirmed GHD in the context of an underlying dysplasia, careful auxological, biochemical, and musculoskeletal monitoring is essential. Finally, establishing a molecular diagnosis can resolve uncertainties, guide family evaluation, and facilitate appropriate genetic counseling regarding the autosomal dominant inheritance of BDA1 and its implications for offspring (Figure 4).

### Patient Perspective

The patient’s father reported an overall positive experience with rhGH therapy. According to the family, treatment was associated with a noticeable improvement in linear growth, increased energy levels, and greater participation in daily activities. Although the requirement for daily injections initially raised concerns, both the patient and his family adapted well to the treatment, which was generally well tolerated.

The onset of a transient unilateral prepubertal gynecomastia caused considerable concern for the patient’s family. Nevertheless, clear communication with the clinical team, together with an appropriate diagnostic evaluation, temporary discontinuation of rhGH, and subsequent successful reintroduction without recurrence, helped reassure the family regarding the safety of continued treatment.

Molecular confirmation of the *IHH* variant provided an etiological explanation for the disproportionate short stature and brachydactyly and enhanced the family’s understanding of the underlying condition and its potential long-term implications. Furthermore, the family reported a high level of satisfaction with the multidisciplinary care provided and emphasized the importance of ongoing medical support, clear communication, and comprehensive genetic evaluation throughout the diagnostic and therapeutic process.

## 4. Conclusions

This case underscores the diagnostic value of genomic testing in children with unexplained short stature and a phenotype suggestive of BDA1, particularly following negative *SHOX* analysis. The coexistence of GHD and an *IHH*-related skeletal dysplasia highlights the importance of integrating genetic and endocrine evaluation, as both conditions may independently contribute to impaired linear growth. In this case, rhGH therapy was associated with a positive auxological response. Transient prepubertal gynecomastia occurred during treatment, representing a rare and reversible adverse event that did not preclude successful treatment continuation.

## Figures and Tables

**Table 1:** Baseline Biochemical and Hormonal Assessment.

Parameter	Result	Reference Range
Hematologic profile		
Hemoglobin	13.2 g/dL	11.5–15.5
Leukocytes	7.4 × 10^9^/L	4.5–13.5
Platelets	312 × 10^9^/L	150–450
General biochemistry and mineral metabolism		
Glucose	87 mg/dL	70–100
Creatinine	0.42 mg/dL	0.30–0.70
AST	24 U/L	<40
ALT	18 U/L	<40
Calcium	9.8 mg/dL	8.8–10.8
Phosphate	5.1 mg/dL	3.5–5.8
Alkaline phosphatase	245 U/L	150–420
Thyroid and adrenal function		
TSH	2.14 mIU/L	0.5–4.5
Free T4	1.21 ng/dL	0.8–1.8
Morning cortisol	14.8 µg/dL	5–25
Celiac disease screening		
Anti-tTG IgA	1.8 U/mL	<10
Total IgA	126 mg/dL	50–250
Gonadal axis		
LH	0.12 IU/L	Prepubertal <0.3
FSH	0.84 IU/L	Prepubertal 0.3–2.5
Testosterone	<0.05 ng/mL	Prepubertal <0.1
GH–IGF axis		
IGF-1	121 ng/mL	80–300
IGF-1 SDS	−0.8	−2.0 to +2.0
IGFBP-3	3.7 mg/L	2.0–5.5

Baseline evaluation was normal, except for subnormal peak GH responses (<7 ng/mL) to clonidine and glucagon, confirming GHD. Abbreviations: ALT, alanine aminotransferase; AST, aspartate aminotransferase; FSH, follicle-stimulating hormone; GH, growth hormone; IGF-1, insulin-like growth factor 1; IGFBP-3, IGF-binding protein 3; LH, luteinizing hormone; SDS, standard deviation score; TSH, thyroid-stimulating hormone; tTG, tissue transglutaminase. Source: own work.

## Data Availability

The original contributions presented in this study are included in the article. Further inquiries can be directed to the corresponding author.

## Abbreviations

The following abbreviations are used in this manuscript:

ACRD	Acrodysostosis
ACMG/AMP	American College of Medical Genetics and Genomics/Association for Molecular Pathology
BDA1	Brachydactyly type A1
BMI	Body Mass Index
CADD	Combined Annotation Dependent Depletion
CD	Celiac disease
CGD	Constitutional growth delay
*FGFR3*	Fibroblast Growth Factor Receptor 3
GH	Growth Hormone
GHD	Growth Hormone Deficiency
IGF-1	Insulin-like Growth Factor 1
IGFBP-3	Insulin-like Growth Factor Binding Protein 3
*IHH*	Indian Hedgehog
MAS	Meconium Aspiration Syndrome
OMIM	Online Mendelian Inheritance in Man
PHP	Pseudohypoparathyroidism
REVEL	Rare Exome Variant Ensemble Learner
rhGH	Recombinant Human Growth Hormone
*SHOX*	Short Stature Homeobox
